# Yield of Two Consecutive Sputum Specimens for the Effective Diagnosis of Pulmonary Tuberculosis

**DOI:** 10.1371/journal.pone.0067678

**Published:** 2013-07-02

**Authors:** Mohammad R. Islam, Razia Khatun, Mohammad Khaja Mafij Uddin, Md. Siddiqur Rahman Khan, Md. Toufiq Rahman, Tahmeed Ahmed, Sayera Banu

**Affiliations:** 1 Centre for Communicable Diseases, International Centre for Diarrhoeal Disease Research, Bangladesh, Dhaka, Bangladesh; 2 Department of Biochemistry and Molecular Biology, University of Dhaka, Dhaka, Bangladesh; McGill University, Canada

## Abstract

**Background:**

From long instances, it is debatable whether three sputum specimens are required for the diagnosis of pulmonary tuberculosis (TB) or TB can be diagnosed effectively using two consecutive sputum specimens. This study was set out to evaluate the significance of examining multiple sputum specimens in diagnosis of TB.

**Methods:**

We retrospectively reviewed the acid-fast bacillus (AFB) smear and culture results of three consecutive days’ sputum specimens from 413 confirmed TB patients which were detected as part of a larger active case finding study in Dhaka Central Jail, the largest correctional facility in Bangladesh.

**Results:**

AFB was detected from 81% (n = 334) patients, of which 89% (n = 297) were diagnosed from the first and additional 9% (n = 30) were from the second sputum specimen. *M. tuberculosis* growth was observed for 406 patients and 85% (n = 343) were obtained from the first sputum and additional 10% (n = 42) were from the second one. The third specimen didn’t show significant additional diagnostic value for the detection of AFB by microscopy or growth of the *M. tuberculosis*.

**Conclusions:**

We concluded from our study results that examining two consecutive sputum specimens is sufficient enough for the effective diagnosis of TB. It can also decrease the laboratory workload and hence improve the quality of work in settings with high TB burden like Bangladesh.

## Introduction

Tuberculosis (TB) remains a global public health malady that claims almost 1.4 million lives annually [1] and a major public health problem in Bangladesh. The World Health Organization (WHO) ranked Bangladesh sixth among the world’s 22 high-burden TB countries, having an estimated prevalence of 411 (188–671)/100,000 population [2]. In 2011, a total of 150,899 cases were detected in Bangladesh, 98,948 of which were sputum smear-positive (SS+) TB cases. The TB mortality rate (45 deaths per 100,000 populations) in Bangladesh is higher than the Southeast Asian region (31 deaths per 100,000 populations) [2]. Before TB can be treated, a diagnosis needs to be made in an efficient and timely manner, preferably at point-of-care (POC), and using accurate and field friendly tools.

The diagnosis of TB is made by identifying the presence of acid-fast bacillus (AFB) under microscopy and/or subsequently culturing *Mycobacterium tuberculosis* (MTB) from sputum or tissue [3]. For the diagnosis of TB, the AFB smear of sputum has traditionally been the first diagnostic test used to screen for active TB disease and most commonly been used by the national TB control programs (NTPs) of different countries including Bangladesh due to limited access to culture facility [4]. The AFB smear can be performed quickly and can provide information to the clinician in less than 24 hours. However, it has several limitations; it is examiner, technique, and prevalence dependent; and it lacks specificity for TB [5], [6]. In clinical practice, patients suspected of having pulmonary TB are placed routinely in TB isolation. Most standard laboratory texts [7] and guidelines for mycobacteriology laboratories [8], [9] recommend that at least three sputum specimens, preferably collected on successive days, be submitted to the laboratory for AFB smear and culture for patients suspected to have TB. Unfortunately, there has been a paucity of published data analyzing the validity of this recommendation [10]. There are studies suggesting that two specimens may be as sensitive as three specimens [11], [12], [13], [14]. Recently, the WHO also recommended reducing the number of specimens to be examined for screening of TB cases from three to two, in places where a well-functioning external quality assurance (EQA) system exists [15]. This would significantly reduce the workload for the laboratories in high-burden countries because less specimens would have to be obtained and processed from each patient to rule out TB. In Bangladesh, the national guideline for TB control program recommends to examine three sputum specimens of which two should be collected in the spot and one in the early morning over two consecutive days.

In the present investigation, we sought to analyze what the overall contribution of each successively collected specimen was to the ultimate diagnosis of pulmonary TB for those patients from whom three sputa were collected to our laboratory. This analysis included the smear and culture results of confirmed TB cases that were identified in an active case finding study in the Dhaka Central Jail, the largest prison facility in Bangladesh. Though several studies confirmed that reduction of the sputum specimen number from three to two is considerably suitable for TB diagnosis at the national level [11], [13], but more studies should be carried out to make the hypothesis established in different country perspective. Therefore our study will strengthen the concept for the reduction of the specimen number in TB diagnosis in Bangladesh.

## Methods

This study was approved by the Ethical Review Committee (ERC) of the International Centre for Diarrhoeal Disease Research, Bangladesh (icddr, b). The study was undertaken in the Dhaka Central Jail, Bangladesh from October 2005 to September 2007, where a staggering high prevalence of TB cases (2,227/100,000 population) was reported [16]. The capacity of the jail is about 2600 including both male and female inmates, but it usually contains about 11,000 inmates at any time. Transmission of TB in such highly dense prison expected to be very high in Bangladesh. The participants were enrolled into the study only after receiving informed written consent and inmates who declined to participate in the study were eligible for treatment and also were not disadvantaged in any other way.

A medical doctor, trained in pulmonology, interviewed the inmates (20–30 inmates/day), examined all TB suspects (having cough for more than three weeks) and collected related clinical and socio-demographic information from the inmates. Only the inmates who did not provide consent to participate into the study were excluded. Three sputum specimens, one in the spot and two in the early morning, were collected from a total of 2,756 suspects and brought to the Tuberculosis Laboratory of icddr,b. We collected three sputum specimens over three consecutive days. The sputum specimens were transported to the laboratory in a cool box maintaining standard laboratory guidelines for TB and processed on the same day for AFB microscopy, culture and drug susceptibility testing by laboratory technicians trained in mycobacteriology. Diagnosis was based upon the demonstration of AFB in sputum samples subjected to Ziehl-Neelsen staining under light microscopy, or growth on culture using Löwenstein-Jensen solid media [5]. A confirmed pulmonary TB case was defined as at least two sputum specimens positive for AFB or at least one sputum specimen showing mycobacterial growth in solid culture media [17]. Sputum smears were decontaminated, digested, and concentrated by standard laboratory methods. Cultures were incubated at 37°C for up to 8 weeks, and isolates of MTB complex were identified by presence of region of difference 9 (RD9) using polymerase chain reaction [18].

Data were entered and analyzed using SPSS 17.0 (SPSS Statistics Data Editor). Comparisons between diagnostic test results were performed using the McNemar test. All reported P values were two-sided.

## Results

In the present study, we concentrated on assessing the contribution of each specimen collected to the ultimate diagnosis of TB for patients with culture-proven disease and from whom at least three sputum specimens had been collected. The enrolled suspects were not diagnosed with TB before and haven’t received any treatment for tuberculosis so far.

In our study, out of 2756 suspected pulmonary TB cases, we obtained 413 confirmed patients combining their AFB smear and culture test. A Socio demographic characteristic of these patients is shown in Table 1. More than 98% of these patients were male and 85% of them have smoking habit.

**Table 1:** Socio demographic characteristics of TB patients.

Characteristics	Sub categories	No. of patients	%
		(n = 413)	
Sex	Male	408	98.8
	Female	5	1.2
Place of residence	Rural	103	24.9
	Urban	310	75.1
Age (y)	10–20	28	6.8
	21–30	221	53.5
	31–40	109	26.4
	more than 40	55	13.3
Occupation	Self-employed	101	24.6
	Business	144	35.0
	Service	123	29.9
	Unemployed	39	9.5
	Housewife	4	1.0
Smoking	Yes	354	85.7
	No	59	14.3
INH prophylaxis		0	0
HIV status		Not tested	

**Figure 1:**
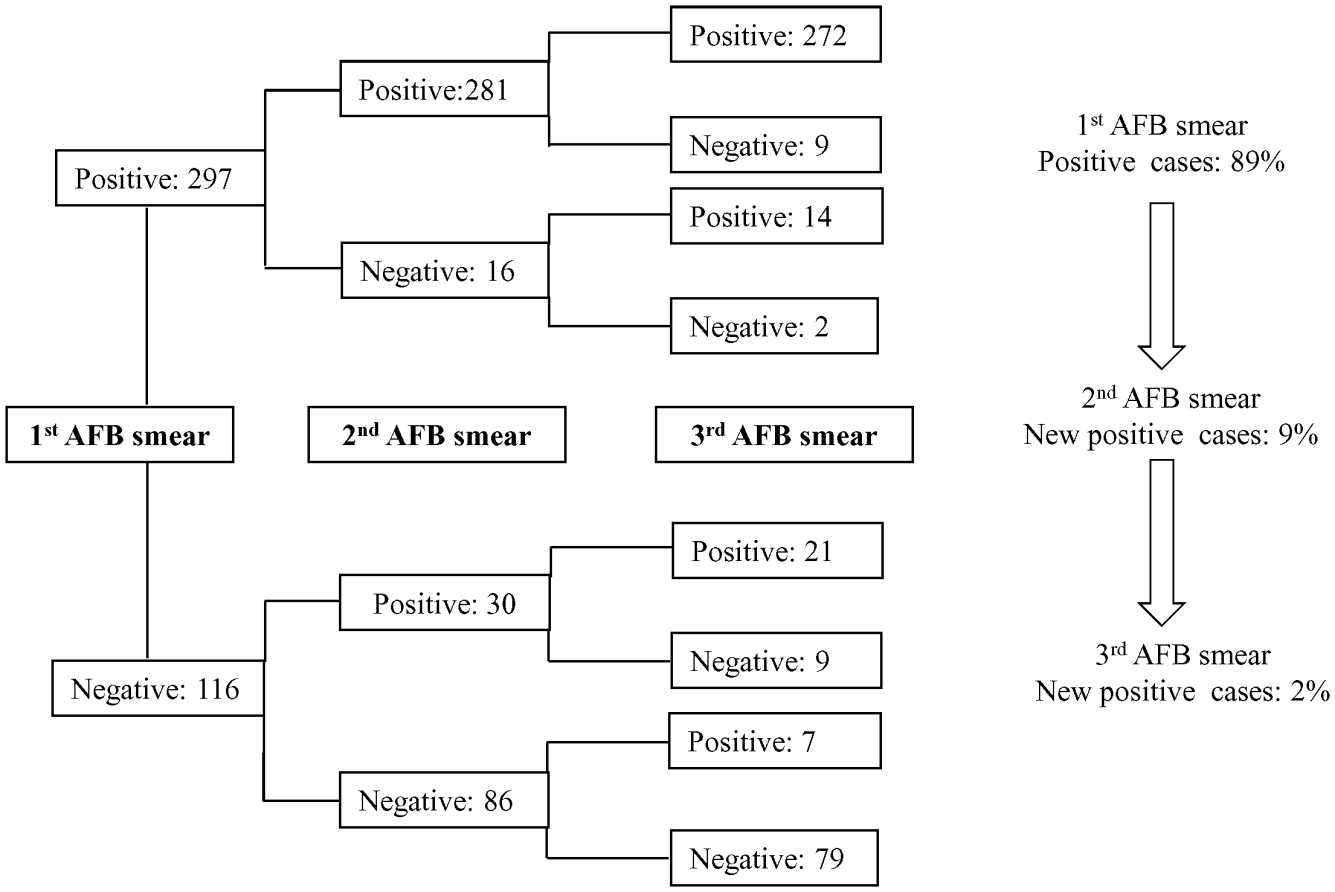
Flow chart for the results of smear examination. The right side one shows the percentage of positive cases (n = 334) added in each smear samples.

AFB was detected from one or more sputum specimens by smear microscopy in 81% (n = 334 of 413) of these confirmed TB cases. Analyzing the results of smear examination of 334 patients showed that 89% (n = 297) were detected from the first smear, additional 9% (n = 30) cases were obtained from the second smear and remaining 2% (n = 7) were from the third. The incremental yield of smear-positive results going from the first, to the second and the third sputum specimen is shown in the Figure 1. The first two samples gave the maximum incremental yield of the TB diagnosis as the third one shows no diagnostic value. Of 413 patients’ sputum specimens, 406 showed growth of *M. tuberculosis* in the culture of which 85% (n = 343) were obtained from the first sputum specimen, additional 10% (n = 42) were from the second and 5% (n = 21) from the third specimen (data not shown). Further analysis was performed with the consecutive sputum specimens from TB patients and their culture results. The total number of culture-positive specimens received for each patient in this group is shown in the Table 2. All three sputum specimens showed culture positivity for 288 patients, of which 258 patients were found smear positive and 30 were smear negative. Seven smear positive patients were unable to produce any growth on culture (all three samples).

**Table 2:** AFB smear result with respect to the number of *M. tuberculosis* culture positive specimens from three consecutive days sputum samples.

No. of culture positive specimens	No. of patients (%) that were:		
	Either^a^	Smear Positive^b^	Smear negative
	(n = 413)	(n = 334)	(n = 79)
0	7 (2)	7 (2)	0 (0)
1	46 (11)	25 (8)	21 (27)
2	72 (17)	44 (13)	28 (35)
3	288 (70)	258 (77)	30 (38)

a Total number of AFB smear positive and negative specimens.

b At least one sequential specimen of the patients was AFB smear positive.

Quality of the sputum should also be considered for the diagnosis of pulmonary TB. Normally in the early morning, good quality sputum can be produced from the TB suspected patients. In our study we found that sputum in the early morning (2^nd^ and 3^rd^ specimen) showed higher detection of AFB than the sputum in the spot (1^st^ specimen). The analysis of the data showed that reliance on the first specimen (spot) could detect 72% (n = 297 of 413) of the sputum smear positive patients (Table 3). In addition, if the second specimen (morning) was also taken into consideration then 75% (n = 311of 413) of the patients could be detected (Table 3). The incremental yield of the third specimen (morning) smear was 1%. If only the early morning specimens were required for testing, then approximately 76 percent of the patients could be detected. This is higher yield than the result with the spot specimen (1st) and the difference is statistically significant (P = 0.01) when we performed the McNemar’s test for comparison of proportions.

**Table 3:** Incremental yield of morning samples in TB case detection.

Sputum specimen	Cases detected	%
	(n = 413)	
First specimen (spot sample)	297	72
Second specimen (morning sample)	311	75
Third specimen (morning sample)	314	76

## Discussion

To control pulmonary TB efficiently, we should develop the strategies for the effective diagnosis of this disease in a cost saving and reducing work load manner. In our study we found that two specimens in consecutive days were as accurate as standard three specimens for the diagnosis of pulmonary TB. The incremental yield of examining a third specimen was very low.

These findings substantially agree with those shown by Nelson et al. [19] and confirm that the first and the second specimens enable *M. tuberculosis* isolation from a majority of patients (98%), while the third or a subsequent specimen collected is of little diagnostic relevance. Similarly, Cascina et al. found the sensitivity of culture increased by 16% with the second specimen and by another 8% with the third specimen [20]. In addition to this document, a recent study involving 42 laboratories in four high-burden countries showed that the incremental yield from a third sequential smear ranged from 0.7 to 7.2% [21]. Our data also strongly indicate that the collection of two sputum specimens is almost always adequate to make a diagnosis. Therefore, the recommendation that three sputum specimens be collected for all patients with suspected TB has limited incremental yield as compared to the first two consecutive specimens and that examination of most of these additional specimens is an inefficient use of laboratory resources. The WHO recommendation for two serial smears (instead of one, as in the Union recommendation) at each follow-up examination may be more problematic, as the prevalence of failures is much lower than the prevalence of cases [22], [23]. Furthermore, the specificity of microscopy in identifying living tubercle bacilli during follow-up is considerably lower than with a diagnostic examination [15]. Of course, once one smear shows any AFB, a confirmatory smear examination should be obtained before declaring an examinee to be a new sputum smear positive case or a bacteriologic treatment failure.

In high-burden settings, the elimination of the third specimen and the resultant reduction in workload may actually improve case detection by improving the quality of examination of the first two specimens. If it were possible to translate the reduction in specimens required into a reduction in the number of patient visits required (e.g., by examining two smears on the same day), case detection and the proportion of all cases treated might be further increased through a reduction in the number of patients who had chance to drop out during the diagnostic process [24]. So, it can be recommended from our study that, for a high burden TB country like Bangladesh, reducing the sputum specimen size by taking two specimens for each patient would be highly effective for TB diagnosis. This will also reduce the cost and workload of the diagnosis in the developing countries. However, as the study was undertaken in a highly prevalent TB setting, to make a general recommendation for policy, further investigation should be carried out in different study settings.
